# Large Language Models in Patient Health Communication for Atherosclerotic Cardiovascular Disease: Pilot Cross-Sectional Comparative Analysis

**DOI:** 10.2196/81422

**Published:** 2026-01-07

**Authors:** Pengfei Li, Yinfei Xu, Xiang Liu, Zhean Shen, Yi Wang, Xinyi Lv, Ziyi Lu, Hui Wu, Jiaqi Zhuang, Yan Chen

**Affiliations:** 1 Department of Emergency and Critical Care Medicine The Affiliated Suzhou Hospital of Nanjing Medical University Suzhou Municipal Hospital, Gusu School, Nanjing Medical University Suzhou, Jiangsu China; 2 Department of Rehabilitation Shandong Provincial Hospital Affiliated to Shandong First Medical University Jinan, Shandong China; 3 Department of Emergency Management School of Health Policy & Management Nanjing Medical University Nanjing, Jiangsu China; 4 Department of Emergency and Critical Care Medicine The First Affiliated Hospital of Nanjing Medical University Nanjing, Jiangsu China

**Keywords:** atherosclerotic cardiovascular disease, large language models, DeepSeek R1, ChatGPT-4o, Gemini, artificial intelligence, AI

## Abstract

**Background:**

Large language models (LLMs) have emerged as promising tools for enhancing public access to medical information, particularly for chronic diseases such as atherosclerotic cardiovascular disease (ASCVD). However, their effectiveness in patient-centered health communication remains underexplored, especially in multilingual contexts.

**Objective:**

Our study aimed to conduct a comparative evaluation of 3 advanced LLMs—DeepSeek R1, ChatGPT-4o, and Gemini—in generating responses to ASCVD-related patient queries in both English and Chinese, assessing their performance across the domains of accuracy, completeness, and comprehensibility.

**Methods:**

We conducted a cross-sectional evaluation based on 25 clinically validated ASCVD questions spanning 5 domains—definitions, diagnosis, treatment, prevention, and lifestyle. Each question was submitted 5 times to each of the 3 LLMs in both English and Chinese, yielding 750 responses in total, all generated under default settings to approximate real-world conditions. Three board-certified cardiologists blinded to model identity independently scored the responses using standardized Likert scales with predefined anchors. The assessment followed a rigorous multistage process that incorporated randomization, washout periods, and final consensus scoring.

**Results:**

DeepSeek R1 achieved the highest “good response” rates (24/25, 96% in both English and Chinese), substantially outperforming ChatGPT-4o (21/25, 84%) and Gemini (12/25, 48% in English and 17/25, 68% in Chinese). DeepSeek R1 demonstrated superior median accuracy scores (6, IQR 6-6 in both languages) and completeness scores (3, IQR 2-3 in both languages) compared to the other models (*P*<.001). All models had a median comprehensibility score of 3; however, in English, DeepSeek R1 and ChatGPT-4o were rated significantly clearer than Gemini (*P*=.006 and *P*=.03, respectively), whereas no significant between-model differences were observed in Chinese (*P*=.08). Interrater reliability was moderate (Kendall *W*: accuracy=0.578; completeness=0.565; comprehensibility=0.486). Performance was consistently stronger for definitional and diagnostic questions than for treatment and prevention topics across all models. Specifically, none of the models consistently provided responses aligned with the latest clinical guidelines for the following key guideline-facing question “What is the standard treatment regimen for ASCVD?”

**Conclusions:**

DeepSeek R1 exhibited promising and consistent performance in generating high-quality, patient-facing ASCVD information across both English and Chinese, highlighting the potential of open-source LLMs in promoting digital health literacy and equitable access to chronic disease information. However, a clinically critical weakness was observed in guideline-sensitive treatment: the models did not reliably provide guideline-concordant standard treatment regimens, suggesting that LLM use should be limited to lower-risk informational subqueries (eg, definitions, diagnosis, and lifestyle education) unless augmented by expert oversight and safety controls.

## Introduction

Atherosclerotic cardiovascular disease (ASCVD) is a major public health concern worldwide, significantly affecting morbidity and mortality rates and contributing to the global burden of disease [1]. Effective management and prevention strategies are paramount in reducing its impact on human health and longevity. In recent years, the development of advanced natural language processing technologies, particularly large language models (LLMs), has introduced a novel paradigm in health care communication [2]. LLMs are deep learning models trained on massive corpora of text data, enabling them to generate humanlike responses, summarize complex information, and interact with users in natural language. Models such as ChatGPT exemplify this technological advancement, offering scalable tools for disseminating medical information and supporting patient education, with the potential to enhance chronic disease prevention and self-management [3,4].

Despite their promise, the application of LLMs in health care—especially in clinical decision-making—remains constrained by several limitations [5,6]. First, LLMs do not inherently possess medical reasoning capabilities; their outputs are generated based on learned language patterns rather than grounded clinical judgment [7]. This introduces risks of producing plausible-sounding but clinically inappropriate advice. Second, the variability in training data sources and lack of domain-specific fine-tuning may result in inconsistent or outdated information [8]. Finally, while individual models may excel in certain tasks, performance can vary widely across different LLMs, especially in medical settings where accuracy and contextual appropriateness are vital [9]. Therefore, rigorous comparative evaluations are needed to identify the models that are most reliable, transparent, and suitable for patient-centered communication within health care and public health contexts.

To our knowledge, there have been no studies specifically focused on the performance of LLMs in providing information about ASCVD. Therefore, this study aimed to assess the performance of various advanced LLMs (ChatGPT-4o [OpenAI], Gemini [Google], and DeepSeek R1) in delivering accurate, comprehensive, and comprehensible information related to ASCVD. Using open-ended questions and simulated patient scenarios, we evaluated the quality and reliability of responses from a patient-centered perspective.

## Methods

### Question Design and Acquisition of Responses

**Figure 1:**
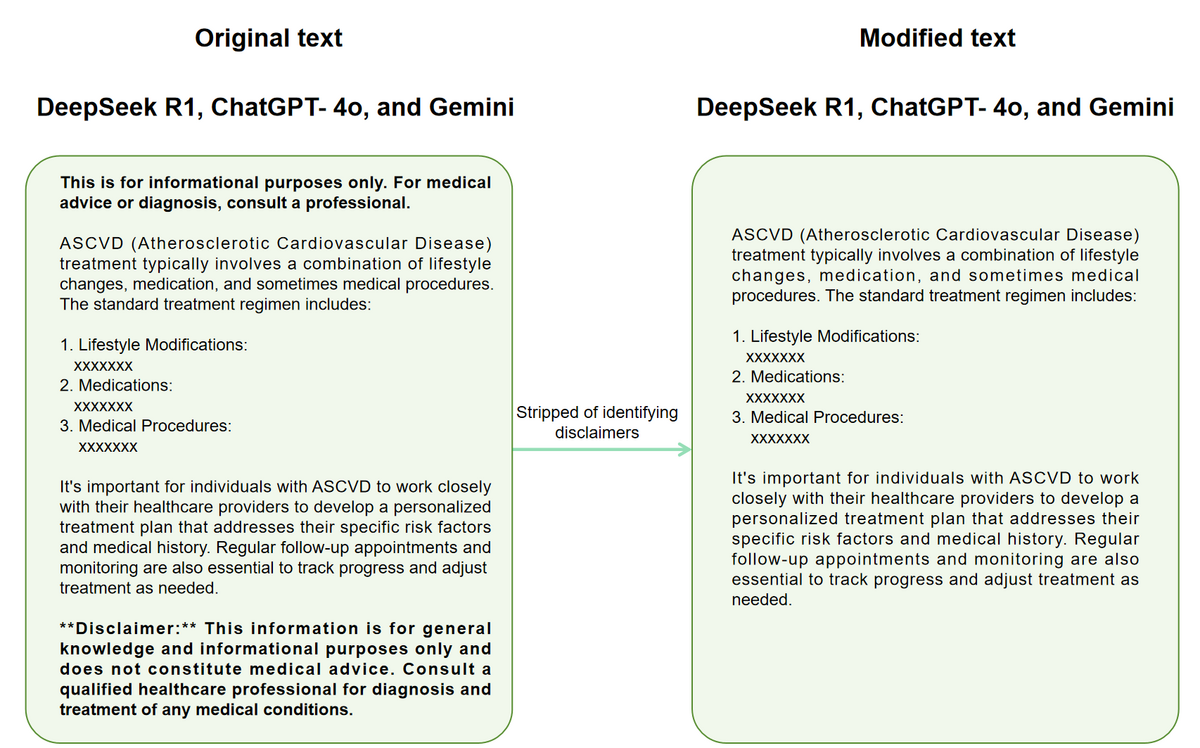
Deidentification and preprocessing of model outputs before blinded scoring: raw large language model responses were exported as plain text and stripped of disclaimers and model-identifying metadata, yielding anonymized text for review by 3 cardiologists.

Our study was conducted from May 15 to 30, 2025. Three qualified physicians (PL, YX, and YW) devised 25 comprehensive questions based on common concerns of patients regarding ASCVD, focusing on patient-centered care and disease management. These queries covered definitions, diagnosis, treatment, prevention, and lifestyle. To mitigate language bias and evaluate multilingual performance, each question was posed in both English and Chinese. To reduce randomness and assess response consistency, each question was submitted 5 separate times to 3 state-of-the-art LLMs—DeepSeek R1, ChatGPT-4o, and Gemini—in both languages, generating a total of 750 responses (25 questions × 3 models × 2 languages × 5 repetitions). To simulate real-world use scenarios, all models were assessed using their default, publicly available versions without any parameter adjustments (eg, temperature, maximum tokens, or top-p nucleus sampling). Crucially, the web-browsing or retrieval-augmented generation capabilities enabled by default in these public interfaces were allowed to function. This ensured that our findings reflected the typical performance accessible to end users who rely on these tools for real-time information retrieval. Each chat was conducted using the “new chat” function to avoid bias from correlation interference and ensure that each session was unaffected by previous prompts. All generated responses were exported as plain text. For DeepSeek R1, the reasoning content and blocks were programmatically removed before human review. Subsequently, all responses were stripped of identifying disclaimers (Figure 1). The complete set of 750 generated responses has been made publicly available in Multimedia Appendix 1 to ensure full transparency and facilitate further research.

### Assessment Methodology

An evaluation panel of 3 board-certified cardiologists assessed the model outputs through a rigorous multistage process. For each of the 25 questions in both languages, 5 independent responses were generated per model. To avoid model selection bias, responses were randomly shuffled within their respective question sets, ensuring that the reviewers remained unaware of the specific language model that generated each response.

To minimize memory bias, 3 cardiologists independently assessed the responses in 3 separate rounds, each conducted on a distinct day with an overnight washout period. Assessments were benchmarked primarily to the 2021 European Society of Cardiology guidelines for cardiovascular disease prevention and the 2024 guidelines for managing chronic coronary syndromes, with cross-checks against the 2025 ACC/AHA/ACEP/NAEMSP/SCAI Guideline for the Management of Patients With Acute Coronary Syndromes [10-12]. We verified that no major guideline updates relevant to our end points were released during the evaluation window (May 15-30, 2025). Using predefined anchors detailed in Table S1 in Multimedia Appendix 2, each reviewer rated every response on 3 dimensions: accuracy (6-point Likert scale: 1=“completely incorrect”; 6=“completely correct”), completeness (3-point Likert scale: 1=“incomplete”; 3=“complete”), and comprehensibility (3-point Likert scale: 1=“difficult to understand”; 3=“easy to understand”) [13]. The scoring protocol comprised 3 steps. First, for each question, each model generated 5 responses; the 3 reviewers independently rated every response on the 3 dimensions. Second, the reviewers convened to finalize one consensus score per response per dimension: identical ratings were carried forward, whereas discrepancies were resolved through discussion until unanimity was reached—or, if needed, with brief input from a senior cardiologist. Third, for each question, the arithmetic mean of the 5 consensus scores served as the final per-question score; because per-question scores were nonnormally distributed, between-platform comparisons used the median of the 25 per-question scores. Detailed scoring procedures for the blinded assessment are provided in Tables S2 to S4 in Multimedia Appendix 2.

**Figure 2:**
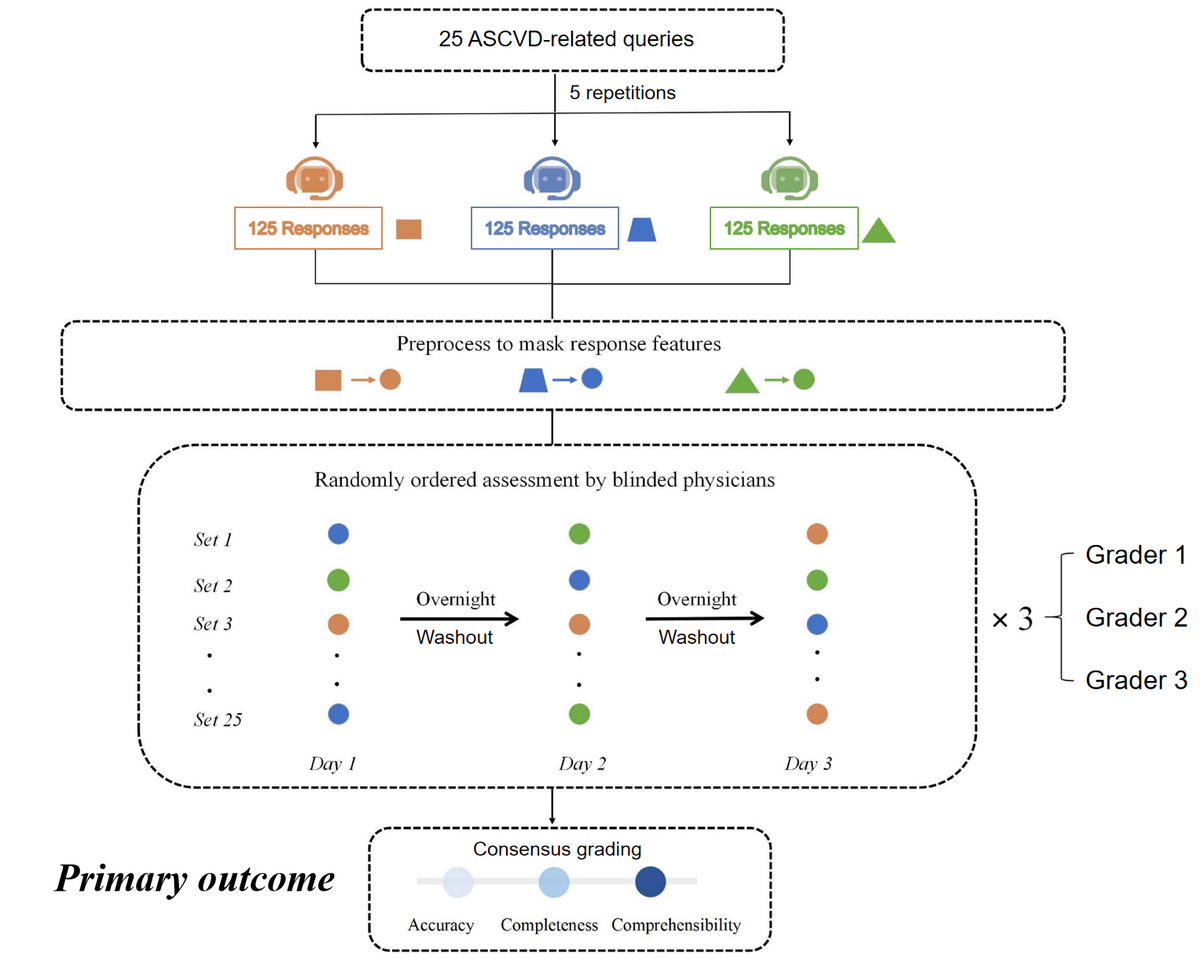
Study design and data flow for the blinded comparison of large language models on atherosclerotic cardiovascular disease (ASCVD) questions. Outputs were generated under default settings; all model-identifying markers and disclaimers were removed; and responses were randomized and presented model blind to cardiologists for independent rating, consensus finalization, per-question aggregation, and cross-model comparison.

The overall study design and process are illustrated in Figure 2. We established the following quality thresholds based on the arithmetic mean of the 5 repeated responses per question: an accuracy score of at least 4 was considered acceptable, whereas a score of at least 2 was required for both comprehensibility and completeness. Within the acceptable performance levels, we introduced a new concept called “good response” to facilitate the comparison of the LLMs’ excellent responses. A “good response” was defined as having a mean accuracy score of ≥4, mean comprehensibility and completeness scores of ≥2, and a mean total score (sum of accuracy, completeness, and comprehensibility) score of ≥10. The total score threshold (10) was intentionally set higher than the sum of the minimum component scores (8) to ensure that a “good response” exceeded the baseline requirements in at least one dimension (eg, higher accuracy or completeness) rather than merely meeting the minimum acceptable standard in all categories.

### Data Analysis

All data analyses were performed using Prism (version 9.5.1; GraphPad Software) and SPSS (version 26.0; IBM Corp). Continuous variables were described using measures of central tendency and dispersion expressed as median and range. Graphical representations were used to enhance the clarity and interpretability of the data. When the data deviated from a normal distribution, differences between 2 groups were assessed using the Mann-Whitney *U* test, whereas differences among the 3 LLMs were evaluated using the Kruskal-Wallis rank sum test followed by the Dunn multiple-comparison test. The Fisher exact test was used for categorical variables. Interrater reliability was assessed using the Kendall *W* coefficient of concordance, with levels of agreement interpreted as follows: poor (0-0.2), fair (0.21-0.4), moderate (0.41-0.6), good (0.61-0.8), and excellent (0.81-1) [14]. A 2-sided *P* value of <.05 was considered statistically significant.

### Ethical Considerations

The data analyzed in our study consisted of deidentified, synthetic outputs generated by LLMs in response to 25 guideline-informed ASCVD questions curated from publicly accessible sources; no patients or personal data were involved. Accordingly, participant recruitment and written informed consent were not applicable, and—per institutional policy—this work did not constitute human participant research and did not require ethics committee review.

## Results

### Performance

#### Overview

**Figure 3:**
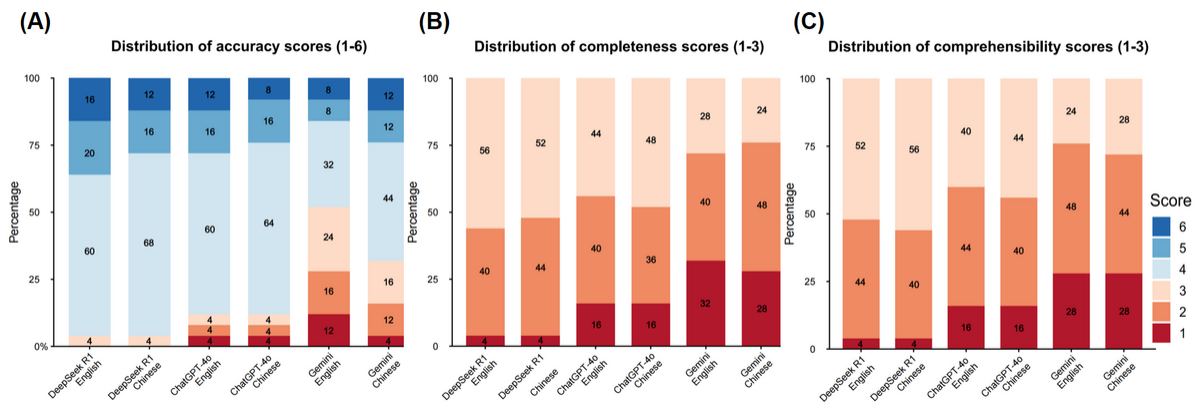
Distribution of accuracy, completeness, and comprehensibility ratings for large language model (LLM) responses to atherosclerotic cardiovascular disease–related questions. (A) Shows the distribution of accuracy scores across LLMs. (B) Shows the distribution of completeness scores across LLMs. (C) Shows the distribution of comprehensibility scores across LLMs.

The “good response” rates for the 3 LLMs were as follows (Table 1): DeepSeek R1 achieved a “good response” rate of 96% (24/25) in both English and Chinese, outperforming ChatGPT-4o (21/25, 84%) and Gemini (12/25, 48% in English and 17/25, 68% in Chinese). Figure 3 illustrates the comparative distribution of scores across the 3 dimensions. In terms of accuracy, DeepSeek R1 maintained a dominant profile with high consistency, whereas Gemini exhibited the widest score variance. Regarding completeness, DeepSeek R1 frequently achieved maximum scores, whereas ChatGPT-4o often provided partially complete responses. Comprehensibility remained high for all models, with DeepSeek R1 and ChatGPT-4o showing slightly more stability than Gemini. Performance analysis revealed a critical pattern: model performance was consistently and substantially weaker in the treatment domain than in all others, with “good response” rates of 80% (8/10) for DeepSeek R1, a total of 60% (6/10) for ChatGPT-4o, and 30% (3/10) for Gemini (Multimedia Appendix 3). For instance, none of the models consistently provided answers that aligned with the latest clinical guidelines in response to the question “What is the standard treatment regimen for ASCVD?” Similarly, the responses varied significantly in accuracy and detail for the question “How common is coronary artery bypass grafting (CABG) in ASCVD patients?” The following sections provide a detailed analysis of each model’s performance across the domains of accuracy, completeness, and comprehensibility.

**Table 1:** Performance of the 3 large language models on atherosclerotic cardiovascular disease (ASCVD) questions in a blinded, bilingual evaluation. Questions spanned 5 domains (definitions, diagnosis, treatment, prevention, and lifestyle) and were scored by 3 board-certified cardiologists using predefined anchors. A “good response” was defined as an arithmetic mean score across the 5 repetitions of ≥4 for accuracy, ≥2 for completeness, and ≥2 for comprehensibility, with a mean total score (sum of accuracy, completeness, and comprehensibility) of ≥10 after consensus.

			DeepSeek English		DeepSeek Chinese		GPT-4o English		GPT-4o Chinese		Gemini English		Gemini Chinese
Good response (N=25), n (%)			24 (96)		24 (96)		21 (84)		21 (84)		12 (48)		17 (68)
**Definitions**													
	“What is ASCVD?”	✓		✓		✓		✓				✓	
	“How does ASCVD develop?”	✓		✓		✓		✓				✓	
	“What diseases does ASCVD specifically include?”	✓		✓		✓		✓		✓		✓	
	“How does ASCVD affect the heart and blood vessels?”	✓		✓									
	“What is the connection between ASCVD and heart disease and stroke?”	✓		✓		✓		✓					
**Diagnosis**													
	“How can I determine my risk for ASCVD?”	✓		✓		✓		✓					
	“What are the early symptoms of ASCVD?”	✓		✓		✓				✓		✓	
	“Which diagnostic tests are used to confirm ASCVD?”	✓		✓		✓		✓					
	“Does a family history of cardiac diseases increase my risk of ASCVD?”	✓		✓		✓		✓		✓		✓	
	“Does my hypertension, diabetes, or hyperlipidemia increase my risk of ASCVD?”	✓		✓				✓				✓	
**Treatment**													
	“What is the standard treatment regimen for ASCVD?”												
	“Is long-term use of lipid-lowering agents safe for individuals with ASCVD?”	✓		✓		✓		✓					
	“Should individuals diagnosed with ASCVD undergo regular coronary angiography?”	✓		✓		✓		✓		✓			
	“What are the nonpharmacological treatments for ASCVD?”	✓		✓		✓		✓		✓		✓	
	“How common is CABG^a^ in ASCVD patients?”	✓		✓									
**Prevention**													
	“Can I prevent ASCVD through diet?”	✓		✓		✓		✓				✓	
	“How important is physical activity in preventing ASCVD?”	✓		✓		✓		✓				✓	
	“Can antihypertensive and lipid-lowering medications prevent ASCVD?”	✓		✓		✓		✓				✓	
	“How effective is smoking cessation in preventing ASCVD?”	✓		✓		✓		✓		✓		✓	
	“How should I monitor my cardiovascular health?”	✓		✓		✓		✓		✓		✓	
**Lifestyle**													
	“With ASCVD, how should I adjust my dietary habits?”	✓		✓		✓		✓		✓		✓	
	“What types of physical activities are safe for someone with ASCVD? Are there any recommended forms of exercise?”	✓		✓		✓		✓		✓		✓	
	“What is the relationship between ASCVD and body weight?”	✓		✓		✓		✓		✓		✓	
	“How important is quitting smoking and reducing alcohol intake in managing ASCVD?”	✓		✓		✓		✓		✓		✓	
	“Can long-term psychological stress affect ASCVD?”	✓		✓		✓		✓		✓		✓	

^a^CABG: coronary artery bypass grafting.

#### Accuracy

Regarding accuracy, 97% (146/150) of the questions met the acceptable standard, with English-language responses reaching 99% (74/75) accuracy and Chinese-language responses reaching 96% (72/75) accuracy. For both English- and Chinese-language questions, the median accuracy score for DeepSeek R1 was consistently 6 (IQR 6-6 for both languages), whereas for ChatGPT-4o and Gemini, it was 5 (ChatGPT: IQR 5-6 for both languages; Gemini: IQR 4-5 for English and 5-6 for Chinese; Table 2). As detailed in Table 3, for all English-language queries, the mean accuracy score of DeepSeek R1 responses was 5.88 (SD 0.33), whereas the mean scores for ChatGPT-4o and Gemini were 5.40 (SD 0.64) and 4.84 (SD 0.68), respectively. For all Chinese-language queries, the mean accuracy score for DeepSeek R1 responses was 5.80 (SD 0.40), whereas the mean ChatGPT-4o and Gemini scores were 5.20 (SD 0.76) and 5.00 (SD 0.86), respectively.

**Table 2:** Median scores for responses from the 3 large language models.

Assessment metric	DeepSeek R1, median (IQR)		ChatGPT-4o, median (IQR)		Gemini, median (IQR)	
	English	Chinese	English	Chinese	English	Chinese
Accuracy (1-6)	6 (6-6)	6 (6-6)	5 (5-6)	5 (5-6)	5 (4-5)	5 (5-6)
Completeness (1-3)	3 (2-3)	3 (2-3)	2 (2-2)	2 (2-2)	2 (2-2)	2 (2-3)
Comprehensibility (1-3)	3 (3-3)	3 (3-3)	3 (3-3)	3 (3-3)	3 (2-3)	3 (3-3)

**Table 3:** Mean scores for responses from the 3 large language models.

Assessment metric	DeepSeek R1, mean (SD)		ChatGPT-4o, mean (SD)			Gemini, mean (SD)	
	English	Chinese	English	Chinese	English		Chinese
Accuracy (1-6)	5.88 (0.33)	5.80 (0.40)	5.40 (0.64)	5.20 (0.76)	4.84 (0.68)		5.00 (0.86)
Completeness (1-3)	2.72 (0.45)	2.76 (0.43)	2.24 (0.43)	2.12 (0.43)	2.00 (0.57)		2.36 (0.63)
Comprehensibility (1-3)	2.96 (0.20)	2.92 (0.27)	2.88 (0.33)	2.84 (0.37)	2.64 (0.48)		2.92 (0.27)

#### Completeness

In terms of completeness, 95% (142/150) of responses were acceptable, including 95% (71/75) in both English and Chinese. Regardless of the language of the questions, the median completeness score for DeepSeek R1 was consistently 3 (IQR 2-3 for both languages), with ChatGPT-4o and Gemini both achieving median scores of 2 (ChatGPT: IQR 2-2 for both languages; Gemini: IQR 2-2 for English and 2-3 for Chinese; Table 2). For all English-language questions, the mean completeness score for DeepSeek R1’s responses was 2.72 (SD 0.45), with ChatGPT-4o and Gemini achieving mean scores of 2.24 (SD 0.43) and 2.00 (SD 0.57), respectively. For all Chinese-language questions, the mean completeness score for responses from DeepSeek R1 was 2.76 (SD 0.43), with ChatGPT-4o and Gemini scoring a mean of 2.12 (SD 0.43) and 2.36 (SD 0.63; Table 3), respectively.

#### Comprehensibility

Regarding comprehensibility, all responses were at an acceptable level. Regardless of the language of the questions or the model used, the median comprehensibility score for all questions was consistently 3 (DeepSeek R1 and ChatGPT: IQR 3-3 for both languages; Gemini: IQR 2-3 for English and 3-3 for Chinese; Table 2). As shown in Table 3, for all English-language questions, the mean comprehensibility score for DeepSeek R1’s responses was 2.96 (SD 0.20), whereas ChatGPT-4o and Gemini scored a mean of 2.88 (SD 0.33) and 2.64 (SD 0.48), respectively. For all Chinese-language questions, the mean comprehensibility score for DeepSeek R1 responses was 2.92 (SD 0.27), with ChatGPT-4o and Gemini scoring a mean of 2.84 (SD 0.37) and 2.92 (SD 0.27), respectively.

### Interrater Reliability

For accuracy scores, the Kendall *W* coefficient of concordance ranged from 0.434 to 0.732, with a mean of 0.578 (SD 0.15). In terms of completeness scores, the coefficient varied from 0.360 to 0.782, averaging 0.565 (SD 0.21). For comprehensibility scores, the range was between 0.386 and 0.581, with a mean value of 0.486 (SD 0.10). This indicates that the scores for accuracy, completeness, and comprehensibility were at a moderate level of agreement.

### Model Comparison

**Figure 4:**
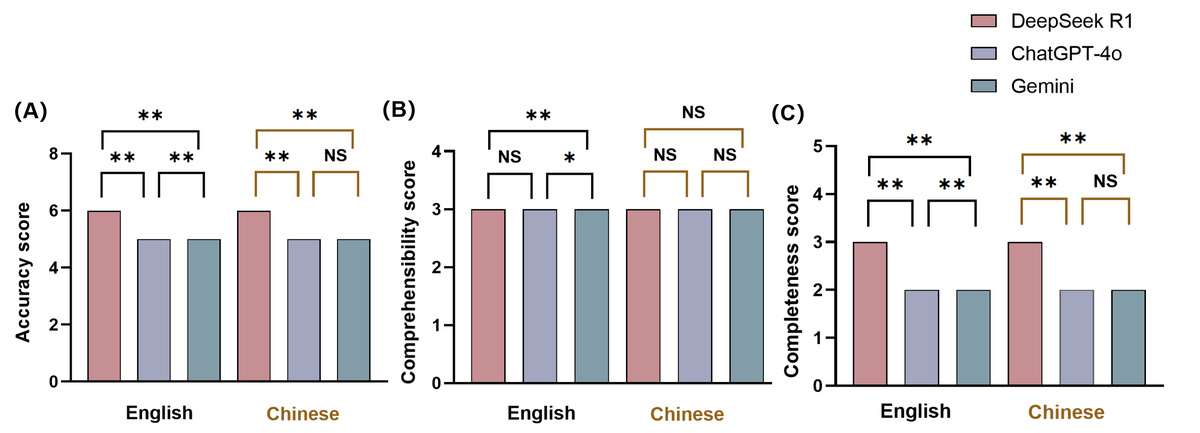
Comparison of large language model performance on atherosclerotic cardiovascular disease–related questions within languages. Scores are shown for accuracy, completeness, and comprehensibility. *P<.05; **P<.01; NS: not significant.

In the overall comparison of English- and Chinese-language questions, DeepSeek R1 consistently outperformed the other models in terms of accuracy and completeness. For English-language questions, DeepSeek R1 achieved the highest median accuracy score of 6, significantly surpassing both ChatGPT-4o and Gemini (*P*<.001 for each pairwise comparison), whereas ChatGPT-4o also outperformed Gemini (*P*=.008). All 3 models received a median comprehensibility score of 3; the difference between DeepSeek R1 and ChatGPT-4o was not significant (*P*=.07), although DeepSeek R1 was significantly clearer than Gemini (*P*=.006), and ChatGPT-4o was moderately clearer than Gemini (*P*=.03; reflecting significant differences in mean ranks per the Mann-Whitney *U* test despite identical medians). Regarding completeness, DeepSeek R1 again led the models, scoring significantly higher than both ChatGPT-4o and Gemini (*P*=.009), with ChatGPT-4o also providing more complete responses than Gemini (*P*=.007). For Chinese-language questions, DeepSeek R1 maintained a significant lead in accuracy over ChatGPT-4o and Gemini (*P*<.001 in both cases), whereas the difference between ChatGPT-4o and Gemini was not statistically significant (*P*=.07). All 3 models achieved the same median comprehensibility score of 3, with no significant differences (*P*=.08). In terms of completeness, DeepSeek R1 again outperformed both ChatGPT-4o and Gemini (*P*<.001), whereas no significant difference was observed between ChatGPT-4o and Gemini (*P*=.06). Overall, DeepSeek R1 demonstrated consistently superior performance in both accuracy and completeness across languages. Differences in comprehensibility were minor, with Gemini showing slightly lower clarity in English. These findings affirm DeepSeek R1’s leading capabilities and highlight subtle language-specific variations in the outputs of the other models (Figure 4). Specific examples of LLM failures contrasted with superior responses are detailed in Table S5 in Multimedia Appendix 2.

### Language-Based Response Comparison

**Figure 5:**
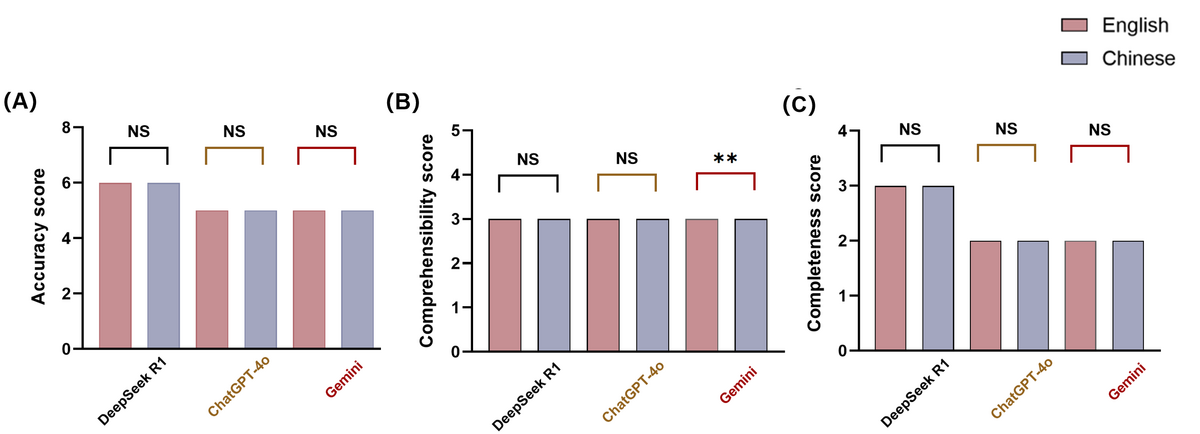
Comparison of large language model performance on atherosclerotic cardiovascular disease–related questions across languages within models. For each model, scores for accuracy, completeness, and comprehensibility are shown for English and Chinese. **P<.01; NS: not significant.

For DeepSeek R1, there were no significant differences between English and Chinese in terms of accuracy, completeness, and comprehensibility scores. Similarly, for ChatGPT-4o, there were no significant differences between English and Chinese across the same metrics, indicating a comparable performance in both languages. For Gemini, the accuracy and completeness scores showed no significant differences between English and Chinese. However, there was a significant difference in comprehensibility scores (*P*<.001) as the median comprehensibility score for English was 3 (IQR 2-3), whereas for Chinese, it was 3 (IQR 3-3). This indicated that the comprehensibility scores were more consistent in Chinese compared to having more variability in English (Figure 5).

## Discussion

### Principal Findings

Our study evaluated the performance of 3 advanced LLMs—DeepSeek R1, ChatGPT-4o, and Gemini—in providing accurate, comprehensive, and comprehensible information on ASCVD in both English and Chinese. This evaluation was conducted using a rigorous, blinded study design that incorporated randomization, washout periods, and consensus-based physician assessments. While the widespread emergence of LLMs has significantly expanded public access to medical information—particularly for underserved or non–English-speaking populations [15,16]—concerns about their reliability persist [17,18]. By systematically evaluating multiple LLMs across languages and using patient-centered ASCVD scenarios, our study offers a more holistic perspective on their capabilities and limitations. From a public health standpoint, this approach not only highlights the potential of LLMs to enhance digital health literacy and self-management of chronic diseases but also underscores the need for ongoing oversight and model validation to prevent the spread of misinformation [19,20].

The results demonstrated that DeepSeek R1 generally outperformed the other models in terms of accuracy and completeness and also achieved a slightly higher average score for understandability. Several factors may contribute to this superior performance. DeepSeek R1 is reported to rely heavily on reinforcement learning, which may contribute to improved logical coherence and multistep reasoning. Additionally, the model is open source under the Massachusetts Institute of Technology license and may offer lower operating costs compared to proprietary models. This open-source nature confers several advantages—including reduced dependency on commercial application programming interfaces (APIs), lower implementation costs, and the potential for institution-specific fine-tuning—making it particularly attractive for health care systems with limited technical resources [21]. Critically, this open-source architecture offers a decisive advantage for clinical implementation: data sovereignty. Unlike proprietary cloud-based models (eg, ChatGPT-4o and Gemini) that necessitate the transmission of sensitive patient data to external commercial servers—raising significant compliance challenges with regulations such as HIPAA (Health Insurance Portability and Accountability Act; United States) and the General Data Protection Regulation (Europe)—DeepSeek R1 allows for local, on-premise deployment [22,23]. Health care systems can host the model within their secure firewalls, ensuring that patient data never leave the institution’s control. This capability not only resolves privacy concerns but also creates a secure environment for the aforementioned fine-tuning on proprietary medical records. Furthermore, it is important to clarify that the high comprehensibility scores in this study were achieved without the reasoning chains being visible to the raters. This indicates that DeepSeek R1’s internal chain-of-thought process effectively structures the final response for clarity independent of the visible reasoning trace [24]. Taken together, these characteristics suggest that DeepSeek R1 may be a promising candidate for disseminating reliable, cost-effective, and customizable medical information in both clinical and public health contexts.

Language-based comparisons revealed no significant performance differences between English and Chinese for both DeepSeek R1 and ChatGPT-4o. Although Gemini exhibited slightly higher scores in Chinese—particularly in comprehensibility—this difference was not statistically significant, indicating that the variation likely reflects incidental model behavior rather than a systematic language-based advantage. Achieving consistent performance across languages is essential for the global applicability of LLMs in health care communication. Our evaluation indicates a consistent and clinically relevant pattern: across all 3 LLMs, recommendations in the treatment and prevention domain were less reliable than responses to definitional and diagnostic queries. This observation is consistent with external reports that therapeutic, guideline-facing tasks show variable concordance and that performance can be highly sensitive to task framing and evaluation metrics beyond headline accuracy [25-27]. Recent benchmarks likewise describe mixed adherence across clinical scenarios, underscoring the difficulty of encoding and operationalizing rapidly evolving therapeutic knowledge within free-text generation [26-28]. Taken together, these findings suggest that, without explicit retrieval and provenance controls, current general-purpose architectures may not consistently reflect the most up-to-date evidence at inference time, which in turn can pose risks in high-stakes areas such as medication dosing and care timing [25-27]. They also help explain why standard offline evaluations may overestimate real-world reliability (“evaluation illusion”) and why even low omission or error rates can have high clinical salience in treatment contexts [9,29].

In practical terms, our findings offer concrete guidance for implementing LLMs in clinical ASCVD care. The encouraging performance of models such as DeepSeek R1 supports adjunctive, nonautonomous use in patient-facing education platforms and, with clinician oversight, selective clinical decision support. For instance, models excelling in comprehensibility are best suited for generating patient education materials, whereas those with superior accuracy on guideline-specific content may better support the drafting of clinical summaries for specialist review. Successful integration will require attention to electronic health record interoperability; comprehensive staff training programs that cover the capabilities, limitations, and appropriate use of these artificial intelligence (AI) tools; and governance (privacy, auditability, and provenance). From a real-world clinical relevance standpoint, cost and deployment considerations (eg, licensing, API use, computing capacity and infrastructure, and local IT constraints) should inform model selection and scaling within existing health care workflows. In particular, open-source options (eg, DeepSeek R1) can lower licensing costs, enable local or hybrid deployment under strict data governance controls, and permit institution-specific tuning with auditable provenance—benefits that may improve feasibility in resource-constrained settings [30]. Given the observed fragility in guideline-sensitive domains, we recommend a clinician-in-the-loop workflow with tiered risk classification, automated guideline-concordance audits, and retrieval-augmented generation to anchor outputs in current evidence, with explicit human sign-off before the information enters care pathways [31,32]. Furthermore, transparent processes for obtaining patient consent for the use of AI-generated content in their care are essential. In addition, institutions should address regulatory and ethical implications (data protection, provenance, and accountability) and establish postdeployment monitoring and incident reporting processes to detect and mitigate potentially harmful responses [33].

### Comparison to Prior Work

Unlike prior studies that have primarily examined single models within narrow clinical contexts [34,35], our investigation extends earlier designs by conducting a head-to-head, bilingual comparison of 3 advanced LLMs—DeepSeek R1, ChatGPT-4o, and Gemini—under a rigorous, blinded methodology incorporating randomization, washout, and consensus-based physician scoring. This design enabled more systematic cross-model comparisons than evaluations focused on 1 system or 1 language. The need for such rigor is underscored by reports from multidisciplinary areas such as cardio-oncology, where ChatGPT has answered only 68% of guideline-based queries correctly [36], highlighting the challenges that general-purpose models face when integrating complex clinical knowledge.

Focusing on ASCVD-related patient education and chronic disease self-management, we found that DeepSeek R1 achieved higher scores for accuracy and completeness and acceptable comprehensibility in both English and Chinese in our dataset, suggesting suitability for patient-facing communication when used nonautonomously. These findings are concordant with recent *Nature Medicine* benchmarks showing that open-source systems can approach the performance of proprietary models in selected tasks [25,37]. Moreover, the open-source nature of DeepSeek R1 may enable privacy regulation–compliant local deployment and institution-specific fine-tuning—features that can support data-governed health systems—whereas its stable bilingual performance in our study aligns with goals of equitable information access [38]. At the same time, our domain-level analysis echoes and extends prior observations by showing that guideline-sensitive content remains comparatively fragile, reinforcing the need for clinician oversight and regular guideline concordance checks [39].

### Limitations

Our study has several methodological limitations that should be considered when interpreting the findings. First, composite constraints in study design, item construction, and the evaluation perspective were present. The question set was clinician led, which may introduce selection bias; it used a fixed, evenly distributed, 5-domain preset (ie, definitions, diagnosis, treatment, prevention, and lifestyle) without direct patient involvement for external validation; model comparison was limited to 3 mainstream LLMs; and assessment relied entirely on expert review without patient-side measures such as comprehension or patient-reported outcomes. Collectively, these factors may affect clinical representativeness, diversity, and ecological validity [40].

Second, we acknowledge a moderate level of interrater agreement among the expert cardiologists as measured using the Kendall *W* (accuracy=0.578; completeness=0.565; comprehensibility=0.486). While such moderate reliability is not uncommon when evaluating nuanced, open-ended medical text where a degree of clinical subjectivity is inherent, it must be noted as a key limitation. To mitigate the impact of this disagreement, we used standardized anchors and a consensus adjudication step, and our statistical analysis (using medians and IQRs with nonparametric tests) was specifically chosen to limit the influence of rater heterogeneity. Nevertheless, this variability introduces uncertainty into the statistical significance of the differences found between models and suggests that expert opinions on AI-generated text quality can vary nontrivially. This is particularly relevant for clinical applications, where the use of such AI-generated content, especially for medical pathways or decision support, demands an even higher degree of caution. Consequently, the findings warrant a cautious interpretation. To enhance reliability in future work, we recommend developing more detailed scoring criteria with concrete examples and implementing a formal rater training and calibration process, including a pilot evaluation to establish baseline agreement.

Third, sample size and statistical power were not determined a priori. As an exploratory benchmarking study in a nascent area lacking established effect sizes, we did not perform an a priori power analysis. We used a coverage and stability-oriented design (25 items across 5 domains and 5 independent generations per model per language; 750 responses in total) to balance breadth and response stability. Although primary between-model contrasts reached statistical significance (*P*<.001)—suggesting adequate sensitivity post hoc—the absence of prespecified power planning limits generalizability; future confirmatory studies should use effect sizes and variance estimates from this work for formal power and sample size planning.

In addition, language and cultural generalizability are limited. Performance was evaluated only in English and Chinese. Differences in health beliefs, communication norms, and linguistic nuance across other languages and cultures may affect acceptability and applicability; broader multilingual, multicultural validation is needed.

Finally, geographic variation and reliance on the chosen reference guidelines (“benchmark binding”) may influence concordance judgments across health systems. While we benchmarked against specific international guidelines, ASCVD management varies by region and health system (eg, drug availability, targets, pathways, and reimbursement). Consequently, judgments of being concordant or nonconcordant are bound to the chosen reference standards, and local calibration is advisable for cross-region application.

### Future Directions

First, longitudinal performance tracking should be prioritized. Standardized benchmarking with regular retesting across model updates, languages, and domains should be established to quantify performance drift, with early warning triggers for degradation; preregistered, multicenter confirmatory studies should be favored.

In addition, direct head-to-head comparisons with human materials should be conducted. LLM outputs should be benchmarked against human-authored patient education materials, assessing quantitative metrics (eg, accuracy and readability) and qualitative outcomes (eg, patient preference and trust) within a composite evaluation framework.

Moreover, pragmatic integration with clinical systems should be pursued. Low-coupling approaches to clinical systems under data minimization and privacy constraints (eg, standardized APIs, compliance and audit trails, and provenance and version management) should be explored to improve deployability.

Furthermore, communication should be tailored to patient literacy, language, culture, and context. Adaptive presentation methods based on health literacy, cultural background, preferred language, and clinical context should be developed and validated, maintaining clinical correctness while improving accessibility and understanding [41]. To ensure that these tailored communication strategies are effective, future work must include patient-centered validation, assessing the correlation between expert ratings and actual patient understanding and incorporating patient-reported outcome measures.

Finally, safety governance should be strengthened with structured risk management and monitoring. Future work should refine scoring anchors and operational definitions, broaden and diversify rater panels with structured calibration, conduct sensitivity and robustness analyses, implement routine guideline concordance audits and a risk stratification framework (eg, dosing errors, contraindications, and care delay advice) [27], and establish continuous monitoring and incident reporting mechanisms to detect and mitigate potentially harmful or misleading outputs [26].

### Conclusions

Our study evaluated the performance of several advanced LLMs in delivering information related to ASCVD, a major chronic disease burden in public health. In a blinded, bilingual comparison of DeepSeek R1, ChatGPT-4o, and Gemini, DeepSeek R1 demonstrated the highest accuracy and completeness, whereas all models showed comparable comprehensibility. Performance was consistently stronger for definitional and diagnostic queries than for treatment and prevention queries, indicating lower alignment with current therapeutic guidance in those domains. Notably, for the core question regarding the standard treatment regimen for ASCVD, none of the models consistently provided answers aligned with the latest clinical guidelines, representing a safety-relevant limitation of current general-purpose LLMs. These results delineate the present capability profile of LLMs for patient information on ASCVD and establish an empirical baseline for subsequent validation.

## Appendix

Multimedia Appendix 1 Full set of large language model responses (N=750; English and Chinese).

Multimedia Appendix 2 Detailed scoring rubric with anchors and examples.

Multimedia Appendix 3 Domain-specific “Good response” rates by model and language.

## Abbreviations

AI: artificial intelligence
API: application programming interface
ASCVD: atherosclerotic cardiovascular disease
HIPAA: Health Insurance Portability and Accountability Act
LLM: large language model

## Data Availability

All data generated or analyzed during this study are included in this published paper and its supplementary information files.
