# Supplementary material for: Large Language Models in Patient Health Communication for Atherosclerotic Cardiovascular Disease: Pilot Cross-Sectional Comparative Analysis
Source: JMIR Med Inform. 2026 Jan 7;14:e81422. doi: 10.2196/81422 (PMC12824577; doi:10.2196/81422)
Supplement: Multimedia Appendix 2 [file medinform_v14i1e81422_app2.docx]

**Table S1. Likert Scales**

| **Score** | **Accuracy rating by a 6-point Likert scale** |
| --- | --- |
| **1** | Completely incorrect |
| **2** | More incorrect than correct |
| **3** | Approximately equally correct and incorrect |
| **4** | More correct than incorrect |
| **5** | Nearly all correct |
| **6** | Completely Correct |
| **Score** | **Completeness rating by a 3-point Likert scale** |
| **1** | Incomplete, addresses some aspects of the question, but significant parts are missing or incomplete |
| **2** | Adequate, addresses all aspects of the question and provides the minimum amount of information required to be considered complete |
| **3** | Comprehensive, addresses all aspects of the question and provides additional information or context beyond what was expected |
| **Score** | **Comprehensibility rating by a 3-point Likert scale** |
| **1** | Difficult to understand |
| **2** | Partly difficult to understand |
| **3** | Easy to understand |

**Table S2. Example of Large Language Model Response Accuracy Evaluation (6-Point Scale)**

| **Prompt**: **What diseases does ASCVD specifically include?** | |
| --- | --- |
| **More correct than incorrect** | **Nearly all correct** |
| ASCVD, or atherosclerotic cardiovascular disease, encompasses a range of conditions that involve the narrowing or blockage of blood vessels due to the buildup of plaque. This can lead to various cardiovascular diseases, including:  1. Coronary artery disease (CAD): Atherosclerosis affecting the arteries that supply blood to the heart muscle.  2. Peripheral artery disease (PAD): Atherosclerosis affecting arteries outside the heart, such as those supplying blood to the limbs, causing reduced blood flow.  3. Carotid artery disease: Atherosclerosis affecting the arteries in the neck that supply blood to the brain, increasing the risk of stroke.  4. Chronic kidney disease (CKD): Atherosclerosis affecting the renal arteries, which can lead to kidney damage and dysfunction.  5. Aortic atherosclerosis: Atherosclerosis affecting the aorta, the body's main artery, which can lead to complications such as aortic aneurysm or aortic dissection.  These diseases are all related to the buildup of plaque in the arteries, leading to reduced blood flow and potentially serious complications like heart attack, stroke, or kidney damage. | Atherosclerotic cardiovascular disease (ASCVD) refers to a spectrum of disorders caused by the accumulation of fatty deposits, or plaque, within arterial walls. This process, known as atherosclerosis, impairs blood flow and can result in several serious conditions. Coronary artery disease arises when the coronary arteries are narrowed, reducing oxygen delivery to the heart. Peripheral artery disease affects vessels in the extremities, often leading to pain or mobility issues. Carotid artery involvement increases the risk of ischemic stroke due to compromised cerebral circulation. Renal artery stenosis, a form of ASCVD, can contribute to chronic kidney disease by limiting blood supply to the kidneys. Additionally, plaque buildup in the aorta may lead to aneurysms or dissections, posing life-threatening risks. Collectively, these manifestations underscore the systemic nature of ASCVD and its potential to cause major organ damage. |
| **Score: Accuracy = 4/6** | **Score: Accuracy = 5/6** |

Accuracy Assessment Based on 6-Point Likert Scale: Response 1 scores 4/6 (More correct than incorrect) for correctly identifying core ASCVD manifestations but inaccurately listing chronic kidney disease as a direct ASCVD entity rather than a complication, while Response 2 scores 5/6 (Nearly all correct ) for precise disease categorization—specifically distinguishing renal artery stenosis from chronic kidney disease—and comprehensively describing the systemic atherosclerotic burden across coronary, peripheral, carotid, and aortic territories, though both responses omit **transient ischemic attacks** per 2021 ESC Prevention and 2024 ESC Chronic Coronary Syndromes guidelines.

**Table S3. Example of Large Language Model Response Completeness Evaluation (3-Point Scale)**

| **Prompt**: **What is the standard treatment plan for ASCVD?** | |
| --- | --- |
| **Incomplete** | **Comprehensive** |
| (ASCVD)is a chronic condition that develops when fatty deposits build up in your arteries (atherosclerosis). This buildup narrows your arteries and reduces blood flow to your organs and tissues. ASCVD can lead to heart attack, stroke, and other serious cardiovascular problems.  There is no one-size-fits-all treatment for ASCVD. The best treatment plan for you will depend on your individual risk factors, such as your age, cholesterol levels, blood pressure, and smoking history. However, there are some general principles that apply to most people with ASCVD.  Lifestyle changes are the cornerstone of treatment for ASCVD. These include: Eating a healthy diet, such as the Mediterranean diet or the DASH diet; Getting regular exercise; Maintaining a healthy weight; Quitting smoking, if you smoke.  Managing stress. Medications can also be used to treat ASCVD. These may include: Statins: Statins are a type of medication that lowers LDL cholesterol, or "bad" cholesterol. | 1. Lifestyle Modifications: Healthy Diet: Emphasizing fruits, vegetables, whole grains, lean proteins, and low-fat dairy while limiting saturated and trans fats, cholesterol, sodium, and added sugars. Regular Exercise: Aim for at least 150 minutes of moderate-intensity aerobic activity or 75 minutes of vigorous-intensity activity per week, along with muscle-strengthening activities on two or more days a week. - Weight Management: Maintaining a healthy weight or losing weight if overweight or obese. Smoking Cessation: Quitting smoking and avoiding exposure to secondhand smoke.  2. Medications: Statins: These are the cornerstone of pharmacologic therapy for ASCVD. They lower LDL cholesterol levels and reduce the risk of cardiovascular events. Examples include atorvastatin, rosuvastatin, and simvastatin. Aspirin: Low-dose aspirin may be recommended for certain individuals to reduce the risk of blood clots. However, aspirin therapy is not suitable for everyone and should be determined based on individual risk factors. Antiplatelet Agents: Besides aspirin, medications like clopidogrel may be prescribed to prevent blood clot formation. Antihypertensive Medications: If blood pressure is elevated, medications such as ACE inhibitors, ARBs, beta-blockers, or calcium channel blockers may be prescribed to lower blood pressure. Other Lipid-Lowering Agents: In some cases, additional medications such as ezetimibe or PCSK9 inhibitors may be added to further lower LDL cholesterol levels.  3. Medical Procedures: Coronary Angioplasty and Stenting: In cases of severe blockages in the coronary arteries, a procedure called percutaneous coronary intervention (PCI) may be performed to open the arteries and place stents to keep them open. Coronary Artery Bypass Grafting (CABG): For individuals with extensive coronary artery disease, bypass surgery may be necessary to create new routes for blood flow to the heart. |
| **Score: Completeness = 1/3** | **Score: Completeness = 3/3** |

Response 1 scores 1/3 (Incomplete) for omitting essential pharmacotherapy beyond statins (**antiplatelet agents, antihypertensives, alternative lipid-lowering drugs**) and all revascularization procedures, while Response 2 scores 3/3 (complete) for comprehensively addressing all guideline-recommended pillars: detailed lifestyle modifications, complete medication classes (statins, antiplatelet therapy, antihypertensives, and additional lipid-lowering agents), and indicated revascularization procedures (PCI and CABG), aligning with 2021 ESC Prevention and 2024 ESC Chronic Coronary Syndromes guidelines.

**Table S4. Example of Large Language Model Response Comprehensibility Evaluation (3-Point Scale)**

| **Prompt**: **What is the connection between ASCVD and heart disease and stroke?** | |
| --- | --- |
| **Partly difficult to understand** | **Easy to understand** |
| Atherosclerotic Cardiovascular Disease (ASCVD) refers to a chronic condition in which fatty deposits, known as plaque, accumulate within the arterial walls. This process, called atherosclerosis, gradually narrows the arteries and restricts blood flow.  1.One major consequence of ASCVD is coronary artery disease (CAD), where the coronary arteries supplying the heart become obstructed. This can result in symptoms like angina, and in severe cases, myocardial infarction or heart failure.  2.Another serious outcome is stroke, which occurs when blood flow to the brain is compromised. Ischemic strokes are typically caused by clots blocking cerebral arteries, while hemorrhagic strokes result from ruptured blood vessels. Atherosclerosis significantly increases the likelihood of ischemic stroke by promoting clot formation.  3.Overall, ASCVD is a progressive vascular disorder that underlies many life-threatening conditions, particularly heart disease and stroke, due to impaired circulation caused by arterial plaque buildup. | Atherosclerotic Cardiovascular Disease (ASCVD) is a broad term that encompasses various heart and vascular conditions resulting from atherosclerosis, which is the build-up of fats, cholesterol, and other substances in and on artery walls (plaque). This buildup can lead to narrowing and blockages in the arteries, diminishing blood flow. The primary conditions under the ASCVD umbrella include coronary heart disease (CHD), such as myocardial infarction (commonly known as a heart attack), and cerebrovascular disease, including strokes. Here’s how ASCVD connects to heart disease and stroke:  1. Connection to Heart Disease: Coronary Artery Disease (CAD): Plaque buildup in the coronary arteries—the major blood vessels that supply blood, oxygen, and nutrients to the heart—can reduce or block blood flow to the heart. This condition is known as CAD, a major component of heart disease. Myocardial Infarction (Heart Attack): If a plaque ruptures, it can form a clot that blocks the flow of blood to heart muscle, causing a heart attack. This is a direct consequence of ASCVD.  2. Connection to Stroke: Carotid Artery Disease: ASCVD can also affect the carotid arteries, which supply blood to the brain. Plaque buildup can either reduce blood flow or cause a clot to form that either partially or completely blocks blood flow to parts of the brain—a primary mechanism of ischemic stroke. Atherothrombotic Stroke: Like heart attacks, strokes can occur when a plaque in the carotid arteries ruptures and forms a clot, leading to a stroke.  3. Risk Factors and Prevention: Common Risk Factors: Both heart disease and stroke share common risk factors that promote ASCVD, including smoking, high blood pressure, high cholesterol, diabetes, obesity, an unhealthy diet, and physical inactivity. Prevention and Management: Managing these risk factors through lifestyle changes (diet, exercise) and medication adherence can significantly reduce the likelihood of developing heart disease or having a stroke due to ASCVD. |
| **Score: Comprehensibility = 2/3** | **Score: Comprehensibility = 3/3** |

Comprehensibility Assessment Based on 3-Point Likert Scale: Response 2 scores 3/3 (easy to understand) for its clear section headings, organized bullet-point structure, and use of patient-friendly language with practical examples, while Response 1 scores 2/3 (Partly difficult to understand) due to its dense paragraph format and more technical terminology, though both accurately convey the relationship between ASCVD, heart disease and stroke.

| **Prompt**: **Should individuals diagnosed with ASCVD undergo regular coronary angiography?** | |
| --- | --- |
| **DeepSeek-R1** | **ChatGPT-4o** |
| **No, regular or routine coronary angiography is NOT recommended for individuals with stable ASCVD.**  Under current international guidelines (such as the ESC and ACC/AHA guidelines), invasive coronary angiography (ICA) is an invasive procedure carrying specific risks. It is **not** a screening tool to be used periodically like a blood test.  **Why is it not done routinely?**  Risks: As an invasive procedure, it carries risks of bleeding, vascular injury, stroke, and reaction to contrast dye. No Clinical Benefit: For patients with stable symptoms on optimal medical therapy, "checking the arteries" just to see the plaque does not change the treatment plan or improve survival.  **When IS it recommended?**  Angiography is typically reserved for specific situations: Worsening Symptoms: If you experience unstable angina (chest pain at rest or with less exertion). Positive Stress Tests: If a non-invasive test (like an ECG stress test or nuclear scan) shows significant ischemia (lack of blood flow). Acute Events: During a heart attack (STEMI/NSTEMI).  **How should I be monitored instead?**  Instead of invasive angiography, your doctor will monitor you using: Risk Factor Control: Regularly checking LDL-cholesterol, blood pressure, and HbA1c. Symptom Assessment: Asking about your exercise tolerance. Non-Invasive Imaging: If needed, a Coronary CT Angiography (CCTA) may be used, which is a scan rather than a surgery, though even this is not done "routinely" without a specific reason. | It depends on your specific condition, but **regular monitoring is a key** part of managing Atherosclerotic Cardiovascular Disease (ASCVD). While lifestyle changes and medications are the first line of defense, your cardiologist might suggest repeating a coronary angiography periodically to keep track of your condition.  The main reason to consider follow-up angiography is to monitor the progression of plaque buildup in your arteries. Over time, ASCVD can progress, and seeing the anatomy of the coronary arteries helps the doctor decide if your current medication is working or if you need an intervention, such as a stent or bypass surgery. Some doctors may prefer to schedule this every few years or if there are minor changes in your health.  However, because it is an invasive procedure involving a catheter, it is not always the first choice for everyone. Sometimes doctors might use other methods first. You should discuss with your healthcare provider to weigh the benefits of seeing the artery blockages against the inconvenience of the procedure to create a personalized schedule for your heart health check-ups. |
| **Total Score: 12/12** | **Total Score: 5/12** |
| **Accuracy Score:6 (Completely Correct)**  **Perfect Guideline Alignment**. The model explicitly stated that routine invasive angiography is not recommended for stable ASCVD, aligning perfectly with current ESC/ACC guidelines (Class III recommendation: Harm/No Benefit). It correctly identified that invasive procedures are reserved for changing symptoms or acute events, avoiding the error of suggesting "preventive screening" via invasive means. | **Accuracy Score:2 (More incorrect than correct)**  **Clinical Error (Over-testing).**The model suggested that angiography might be performed "periodically to track plaque progression." This is clinically incorrect and dangerous advice. Invasive angiography is not used for longitudinal monitoring of plaque in stable patients due to procedural risks; suggesting otherwise constitutes "over-testing." |
| **Completeness Score:3 (Comprehensive)**  **Exceeded Expectations.**Beyond answering "No," the model provided critical context: it explained *why* (procedural risks vs. lack of benefit), listed specific *indications* for when it is actually needed, and offered *non-invasive alternatives* (e.g., CCTA, risk factor control), thus providing a holistic management perspective. | **Completeness Score:1 (Incomplete)**  **Missing Safety Information.**While addressing the prompt, the response failed to mention significant procedural risks (e.g., bleeding, stroke, contrast nephropathy) associated with invasive angiography. It also failed to clearly distinguish between invasive catheterization and non-invasive imaging, which is a critical distinction for patient consent. |
| **Comprehensibility Score:3 (Easy to understand)**  **High Readability.** The response utilized clear headings ("Why," "When," "Alternatives"), bullet points, and bold text. The directness of the answer ("No, it is not recommended") reduced cognitive load for the patient. | **Comprehensibility Score:2 (Partly difficult to understand)**  **Vague/Hedging.**The response relied on dense paragraphs and ambiguous language ("It depends," "might suggest," "some doctors"). This "hedging" makes it difficult for a patient to determine the correct course of action and obscures the medical consensus against routine invasive testing. |

**Table S5.** **Examples of Large Language Models Failure Modes Contrasted with Superior Responses**
