# Supplementary material for: Large Language Models in Patient Health Communication for Atherosclerotic Cardiovascular Disease: Pilot Cross-Sectional Comparative Analysis
Source: JMIR Med Inform. 2026 Jan 7;14:e81422. doi: 10.2196/81422 (PMC12824577; doi:10.2196/81422)
Supplement: Multimedia Appendix 3 [file medinform_v14i1e81422_app3.docx]

**Multimedia Appendix 3**

**Domain-specific “Good response” rates by model and language in a blinded comparative evaluation of three LLMs on 25 ASCVD patient-centered questions.**

| ***Model*** | ***Language*** | ***Domain: Definition*** | ***Domain: Diagnosis*** | ***Domain: Treatment*** | ***Domain: Prevention*** | ***Domain: Lifestyle*** |
| --- | --- | --- | --- | --- | --- | --- |
| ***DeepSeek-R1*** | *English* | 5 (100%) | 5 (100%) | 4 (80%) | 5 (100%) | 5 (100%) |
|  | *Chinese* | 5 (100%) | 5 (100%) | 4 (80%) | 5 (100%) | 5 (100%) |
|  | ***Total*** | **10 (100%)** | **10 (100%)** | **8 (80%)** | **10 (100%)** | **10 (100%)** |
| ***ChatGPT-4o*** | *English* | 4 (80%) | 4 (80%) | 3 (60%) | 5 (100%) | 5 (100%) |
|  | *Chinese* | 4 (80%) | 4 (80%) | 3 (60%) | 5 (100%) | 5 (100%) |
|  | ***Total*** | **8 (80%)** | **8 (80%)** | **6 (60%)** | **10 (100%)** | **10 (100%)** |
| ***Google Gemini*** | *English* | 1 (20%) | 2 (40%) | 2 (40%) | 2 (40%) | 5 (100%) |
|  | *Chinese* | 3 (60%) | 3 (60%) | 1 (20%) | 5 (100%) | 5 (100%) |
|  | ***Total*** | **4 (40%)** | **5 (50%)** | **3 (30%)** | **7 (70%)** | **10 (100%)** |
